# USP38 regulates the stemness and chemoresistance of human colorectal cancer via regulation of HDAC3

**DOI:** 10.1038/s41389-020-0234-z

**Published:** 2020-05-13

**Authors:** Wei Zhan, Xin Liao, Jing Liu, Tian Tian, Lei Yu, Rui Li

**Affiliations:** 1 Department of Colorectal Surgery, Affiliated Hospital of Guizhou Medical University, 550004 Guiyang, Guizhou China; 2 Department of Imaging, Affiliated Hospital of Guizhou Medical University, 550004 Guiyang, Guizhou China; 3 Department of Pathophysiology in Basic Medical College, Guizhou Medical University, 550004 Guiyang, Guizhou China; 4 Department of Pathology, Guiyang Maternal and Child Health Hospital, 550002 Guiyang, Guizhou China; 5 Department of Traditional Chinese Medicine, Guizhou Provincial People’s Hospital, 550002 Guiyang, Guizhou China

**Keywords:** Cancer, Cell biology

## Abstract

Histone modification represents a crucial level of gene expression regulation and is actively involved in the carcinogenesis of human colorectal cancer. Histone acetyltransferases and deacetylases modulate the landscape of histone acetylation, which controls key genes of colorectal cancer pathology. However, the fine tune of histone deacetylases, especially the modification of histone deacetylases that facilitate colorectal cancer, remains elusive. Here, we identified that an ubiquitin-specific protease (USP), USP38, was downregulated in clinical colorectal cancer samples and colorectal cancer cell lines. Importantly, our results showed that USP38 was a specific deubiquitinase of histone deacetylase 3 (HDAC3), which cleaved the lysine 63 ubiquitin chain. Ubiquitination of HDAC3 resulted in a decreased level of histone acetylation and finally led to upregulation of cancer stem cell-related genes. In addition, our results demonstrated a tumor suppressor role of USP38 in colorectal cancer via inhibiting cancer stem cell populations. Most importantly, the ubiquitination level of HDAC3 was responsible for USP38 mediated regulation of cancer stem cell-related transcripts. Our data provided functional insights of USP38 and HDAC3 in colorectal cancer and revealed novel mechanisms of ubiquitination mediated epigenetic regulation.

## Introduction

Histone modification is a vital epigenetic regulatory in the development of colorectal cancer^1^. Recently, single cell based multi-omics sequencing have revealed a histone modification landscape of colorectal cancer patient, which further demonstrated the critical role of epigenetic regulation of gene expression in colorectal carcinogenesis^2^. Histone modifications define the status of certain loci and transcription factors and other DNA binding proteins that usually require active histone modification for transcription of certain genes^3^. Major histone modifications include acetylation, methylation, phosphorylation, SUMOylation, and ubiquitination^1,4^. Acetylation of lysine in histone has been well-characterized and plays crucial functions in colorectal carcinogenesis^5,6^.

Histone acetylation is a reversible and active process which requires histone acetyltransferases and histone deacetylases^7^. Histone deacetylase 3 (HDAC3) was initially identified as a tether of the promoters and ubiquitously expressed in various cell types^8^. It has been demonstrated that HDAC3 is upregulated in human colon tumors and regulates colon cell maturation^9^. However, later study showed that inhibition of HDAC3 had no effect on the growth of colorectal cancer cells^10^. Importantly, previous study have shown that Polycomb chromobox proteins 4 recruits HDAC3 to the promoter of Runx2 and suppresses metastasis of colorectal cancer^11^. These studies suggested that the modification mediated by HDAC3 rather than the expression level of HDAC3 is crucial to carcinogenesis of colorectal cancer. Reports have shown that HDAC3 is degraded by ubiquitin E3 ligase seven in absentia homolog 2 (SIAH2) and ubiquitin like with PHD and ring finger domains 1 (UHRF1)^12,13^. Interestingly, PIWI protein was found to be involved in the modifications of HDAC3^14^. However, the mechanism by which HDAC3 was recruited to specific promoters that plays critical roles in colorectal cancer is largely unknown. Moreover, it is also unclear how ubiquitination of HDAC3 modulates its function.

Ubiquitin-specific protease 38 (USP38) was originally characterized as a negative regulator of type I interferon signaling that regulates TANK binding kinase 1 (TBK1) ubiquitination^15^. Recently, USP38 has been shown to be involved in asthmatic pathogenesis^16^. Moreover, study has indicated that USP38 stabilizes protein lysine-specific histone demethylase 1A (LSD1), a crucial histone modifier, by cleaving its ubiquitin chain^17^. However, the role of USP38 in cancer is still under investigation.

Here, out data showed that UPS38 is significantly down-regulated in colorectal cancer cells and clinical colorectal cancer tissues. USP38 functions as a tumor suppressor through modulating the ubiquitination of HDAC3, which further controls the expression of cancer stem cell-related genes. Hence, our data illustrate that USP38 mediated epigenetic regulation of oncogenes inhibits colorectal tumorigenesis, which provides evidences of HDAC 3 mediated chemoresistance.

## Materials and methods

### Cell lines and cell culture

Human colorectal cancer (CRC) cell lines were purchased from American Type Culture Collection (ATCC; Manassas, VA). SW620, SW480 cell lines were cultured in Leibovitz’s L-15 Medium (Gibco, USA). The culture medium for HCT116, HT29, and human colon epithelial cell line NCM460 was McCoy’s 5a (Gibco, USA). Human colon epithelial cell line FHC was cultured in DMEM: F12 (Gibco, USA) medium, and HCoEpiC was cultured in RPMI1640 (Gibco, USA). All cell lines were maintained in basal medium supplemented with 10% fetal bovine serum (FBS, Gibco, USA), 100 units/mL penicillin and streptomycin (Invitrogen, UK). The reagents used to inhibit DNA methylation and histone deacetylation were 5-Aza-2-deoxycytidine (5 mmol/L, 72 h) and Trichostain A (600 nmol/L, 24 h). 5-fluorouraci (F6627) and oxaliplatin (O9512) were from Sigma-Aldrich. 5-Aza-2-deoxycytidine (S1200), Trichostatin A (TSA) (S1045), MG132 (S2619), and RGFP966 (S7229) was from Selleck.

### Patients and sample collection

All the human colorectal cancer specimens were collected in Affiliated Hospital of Guizhou Medical University. The use of patient samples was approved by the Ethics Committee of Guizhou Medical University (Approval no. 2018–123–01) and informed consent was obtained from the patients. For Cox survival analysis, the patients were classified according to *USP38* median expression level.

### Immunohistochemistry (IHC)

Formalin-fixed, paraffin-embedded (FFPE) tissues were obtained from Affiliated Hospital of Guizhou Medical University and patients enrolled were informed of the scientific usage of the samples. Routine IHC were performed as previously described^18^. USP38 antibody (17767-1-AP) was purchased from Proteintech.

### Plasmid constructs and cell infection

To construct stable overexpression cell lines, full length USP38 were cloned into pLenti-EF1a-Puro-CMV-MCS. To construct stable knockdown cell lines, two different shRNA sequences target USP38 and a non-silencing shRNA sequence were cloned into pLKO-Puro. The sequences of shRNA are as follows: shUSP38–1: SenseSeq: GGGUAAUUGCACUCCUGAAtt, AntiSeq: UUCAGGAGUGCAAUUACCCat; shUSP38–2: SenseSeq: GGUCUUAUUAACCUAGGAAtt, AntiSeq: UUCCUAGGUUAAUAAGACCag. Lentivirus production and infection were generated as previously described in 293T cells^19^. 293T cells were seeded at 105 cells transfected with plasmids using Lipofectamine 2000 (Invitrogen). Viral supernatant was harvested 48 h after transfection. Cells were infected for 12 h and cultured for another 24 h and collected.

### Tumor xenografts

Six-week-old female BALB/c nude mice were purchased from Shanghai Laboratory Animal Center, Chinese Academy of Sciences and Technology (Shanghai, China). All animals were housed and maintained in specific pathogen-free conditions according to the recommendation of Guide for the Care and Use of Laboratory Animals of the National Institutes of Health with strict accordance with protocols approved by the Institutional Animal Care and Use Committee of Guizhou Medical University.

For tumor xenograft model, 2 × 10^6^ indicated HCT116 cells were injected subcutaneously on the right side of the dorsum (*n* = 6 for each group). The tumor diameters were measured every 7 days. Tumor volumes were calculated with the formula: *V* = 1/2 × A × B2. The mice were sacrificed after 6 weeks and tumors were excised.

### Cell growth curve and colony formation assay

For cell growth curve, 1 × 10^4^ cells were seeded in each well of 12-well plate. Cell numbers were counted every 24 h for 5 days. For colony formation assays, 500 indicated cells were seeded in each well of a six-well plate and cultured for 10 days. The colonies were fixed and stained with crystal violet.

### RNA extraction and quantitative PCR (qPCR)

Colorectal cancer cells were collected by centrifugation at 300×*g* for 3 min. Cells were lysed directly with TRIzol reagent (Invitrogen). Total RNAs were extracted according to the manufactory’s protocol and reverse transcribed to with a high-capacity cDNA kit (TaKaRa). qPCR for target genes was performed using SYBR Green (TaKaRa). At least three biological replicates were performed, and each experiment was performed with triplicate or quadruplicate PCR reactions. Data were expressed using the comparative cycle threshold method. Primers used were in Table 1.

**Table 1:** Primer sequences.

Gene	Forward primer (5′-3′)	Reverse primer (5′-3′)
SOX2	CCTCCGGGACATGATCAGCATGTA	GCAGTGTGCCGTTAATGGCCGTG
CD133	CTGGGGCTGCTGTTTATTATTCTG	ACGCCTTGTCCTTGGTAGTGTTG
NANOG	TTTGTGGGCCTGAAGAAAACT	AGGGCTGTCCTGAATAAGCAG
OCT4	ATTCAGCCAAACGACCATCT	GTTTTCTTTCCCTAGCTCCTCC
BIM1	CTGGTTGCCCATTGACAGC	CAGAAAATGAATGCGAGCCA
SNAIL	TCGGAAGCCTAACTACAGCGA	AGATGAGCATTGGCAGCGAG
ABCG2	CAGGTGGAGGCAAATCTTCGT	ACCCTGTTAATCCGTTCGTTTT
CD44	TGGAGCAAACACAACCTCTG	TCCACTTGGCTTTCTGTCCT
USP38	CAGTGCGAGGCCATGTTTG	CGGTGGTATCGTGCGTAGG
GAPDH	GGAGCGAGATCCCTCCAAAAT	GGCTGTTGTCATACTTCTCATGG

### mRNA half-life test

Colorectal cancer cells were seeded at confluence of 60% and treated with 5 mg/mL actinomycinD (Sigma), and RNA was extracted at the indicated time and analyzed with real-time PCR.

### Chromatin Immunoprecipitation (ChIP)

Cell lysates were cross-linked with 1% formaldehyde for one hour at room temperature, and quenched with 125 mmol/L glycine. The nuclear extracts were sonicated and incubated with IgG or anti acetylated H3K27 antibody. Protein-DNA complexes were immunoprecipitated and washed. DNA were then released and captured with the silica membrane purification kit (TIANGEN). qPCR analyses were performed to determine the immunoprecipitated genomic DNA. Primers used for qPCR analysis of CD133 promoter region were as follow: F: 5′-GGTGAGTGTGCGAACTGGAC-3′, R: 5′-TCTTGCCAGAGAGAAGGGGT-3′.

### Flow cytometry analysis

For the analysis of CD44 positive and CD133 positive cells, cells were stained with PE-conjugated anti-CD133 (Miltenyi Biotec) and APC-conjugated anti-CD44 (BD) antibody.

For apoptosis analysis, cells were stained with Annexin V-FITC Apoptosis Kit (K101, Biovision, Milpitas, CA, USA) according to the manufacturer’s instructions.

After the indicated labeling, cells were analyzed with MoFlo Astrios (Beckman-Coulter, CA, USA).

### Western blot and immunoprecipitation (IP)

A full 10-cm plate of cells were collected for each IP or western blot experiment. Cells were lysed on ice in IP buffer (20 mM Hepes (pH 7.4), 0.5% Triton-X-100, 150 mM NaCl, 1.5 mM MgCl) for IP experiments or RIPA buffer (Solarbio) for western-blot experiments for 30 min and the cell lysate was centrifuged for supernatant protein collection. The proteins from cell extracts were immunoprecipitated out using protein A-Sepharose (GE) and specific antibodies. Western blot was carried out using standard procedures, and immune-reactive proteins were visualized by SuperSignal™ chemiluminescence (Thermo Scientific). Primary antibodies against CD133 (#64326), CD44 (#37259), SOX2 (#3579), OCT4 (#2840), SNAIL (#3879) and BMI1 (#5856) were purchased from Cell Signaling Technology. Antibodies against β-actin (A5316), Ubiquitin (AB1690), Lys48-Specific ubiquitin (05–1307), Lys63-Specific ubiquitin (05-1308) and Acetyl-Histone H3 (Lys27) (17–683) were purchased from Sigma-Aldrich. Antibodies against USP38 (17767-1-AP), HDAC1 (1019701-AP), HDAC2 (12922-3-AP), HDAC3 (10255-1-AP), HDAC4 (66838-1-lg), HDAC8 (17548-1-AP) and Histone-H3 (17168-1-AP) were from Proteintech. Antibody against ABCG2 (sc-25822) was from Santa Cruz Biotechnology.

### HDAC activity analysis

The activities of histone deacetylase were analyzed with Histone Deacetylase (HDAC) Activity Assay Kit (Fluorometric) (ab156064, abcam) according to the manufactory’s protocol. Briefly, 1 × 10^7^ cells were washed and resuspended in lysis buffer (10 mM Tris HCl (pH 7.5), 10 mM NaCl, 15 mM MgCl_2_, 250 mM Sucrose. 0.5% NP-40, 0.1 mM EGTA) and lysed on ice for 15 min. Cells were centrifuged through 4 mL of sucrose cushion at 1300 × *g* for 10 min at 4 °C. Pellet were washed and resuspended in extraction buffer (50 mM Hepes KOH (pH 7.5), 420 mM NaCl, 0.5 mM EDTA Na2, 0.1 mM EGTA, 10% glycerol) and then sonicated. Nuclei were lysed for 30 min and centrifuged at 20,000 × *g* for 30 min. Took the supernatant and stored at −80 °C. Reaction wells of a 96-well plates were prepared according to the manufactory’s protocol. Measure fluorescence intensity at 2 min intervals at Ex/Em = 355/460 nm.

### Oncosphere formation and culture

Cells were cultured as oncospheres in culture mediums (described above) supplemented with 10 ng/mL recombinant human basic fibroblast growth factor (R&D Systems), 20 ng/mL recombinant human epidermal growth factor (Promega), 4 mg/mL heparin sulfate (Sigma) and B27 supplement. Thousand cells were seeded in each well of a 6-well ultra-low attachment plates. After 2 weeks of culture, spheres with diameters larger than 50 mm were counted. Oncospheres were digested with 0.25% trypsin and resuspended to seed in new plates as described above.

### Statistical analyses

Statistical analyses were performed with GraphPad Prism 5.0. (GraphPad Software). Experiments were performed at least in triplicates and error bars stands for S.D. Two-tailed Student’s *t*-test was performed to determine the significance of paired data. One-way analysis of variance (ANOVA) for quantitative data from grouped DataSets. *P* value < 0.05 was considered significant. Asterisks indicates *P < 0.05; **P < 0.01, and ***P < 0.001. A log-rank test was performed to compare tumor-free survival. *P* values less than 0.05 were considered statistically significant.

## Results

### USP38 is downregulated in human colorectal cancer

**Fig. 1:**
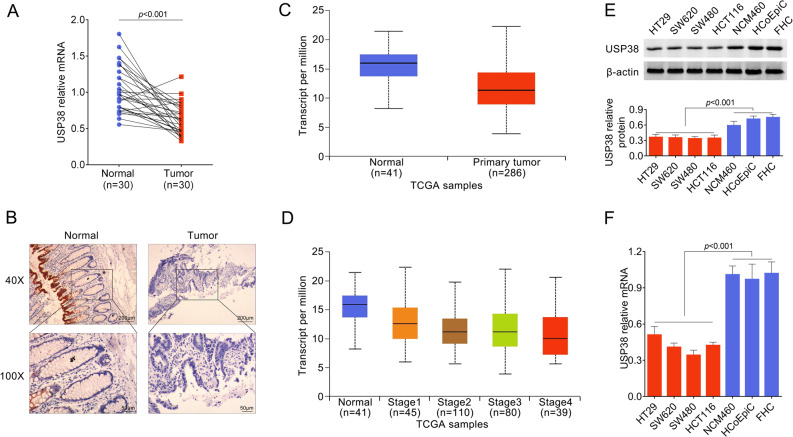
USP38 is downregulated in human colorectal cancer. **a** mRNA level of USP38 in 30 pairs of colorectal cancer samples and respective adjacent tissues. **b** Representative immunohistochemistry (IHC) images of USP38 staining in colorectal cancer samples and respective adjacent tissues. **c** Expression levels of USP38 in colorectal cancer tissues and normal colon tissues. Data were collected from TCGA database. COAD stands for colorectal adenocarcinoma. **d** Expression levels of USP38 in normal colon tissues and colorectal cancer tissues of different stages. Data were collected from TCGA database. COAD stands for colorectal adenocarcinoma. **e** Protein levels of USP38 in four colon cancer cell lines (HT29, SW620, SW480, and HCT116) and three normal colonic epithelial cell lines (NCM460, HCoEpiC, and FHC). The lower panel is quantification of western bolt results. **f** mRNA levels of USP38 in four colon cancer cell lines (HT29, SW620, SW480, and HCT116) and three normal colonic epithelial cell lines (NCM460, HCoEpiC, and FHC).

To evaluate the role of USP38 in colorectal cancer, we first analyzed the expression levels of USP38 in clinical colorectal cancer samples and respective adjacent tissues. Our results showed that both mRNA levels and protein levels of USP38 were significantly decreased in colorectal cancer samples compared to adjacent tissues (Fig. 1a, b) suggesting a tumor suppressive role of USP38 in human colorectal cancer. Additionally, we analyzed the data in The Cancer Genome Atlas (https://www.cancer.gov/about-nci/organization/ccg/research/structural-genomics/tcga) database and found that the transcripts of UPS38 were decreased in primary colorectal tumors in comparison to normal colorectal tissues (Fig. 1c). Interestingly, further analysis of this batch of samples revealed that the expression levels of USP38 decreased as malignancy grade increases (Fig. 1d). We next examined the expression levels of UPS38 in multiple colon cancer cell lines and normal colonic epithelial cells. As expected, the expression levels of UPS38 were significantly decreased in four colon cancer cell lines (HT29, SW620, SW480, and HCT116) in comparison to three normal colonic epithelial cell lines (NCM460, HCoEpiC, and FHC) at both protein levels (Fig. 1e) and mRNA levels (Fig. 1f). Hence, our data illustrated that USP38 is downregulated in human colorectal cancer.

### USP38 inhibits colorectal cancer cell growth in vivo and in vitro

**Fig. 2:**
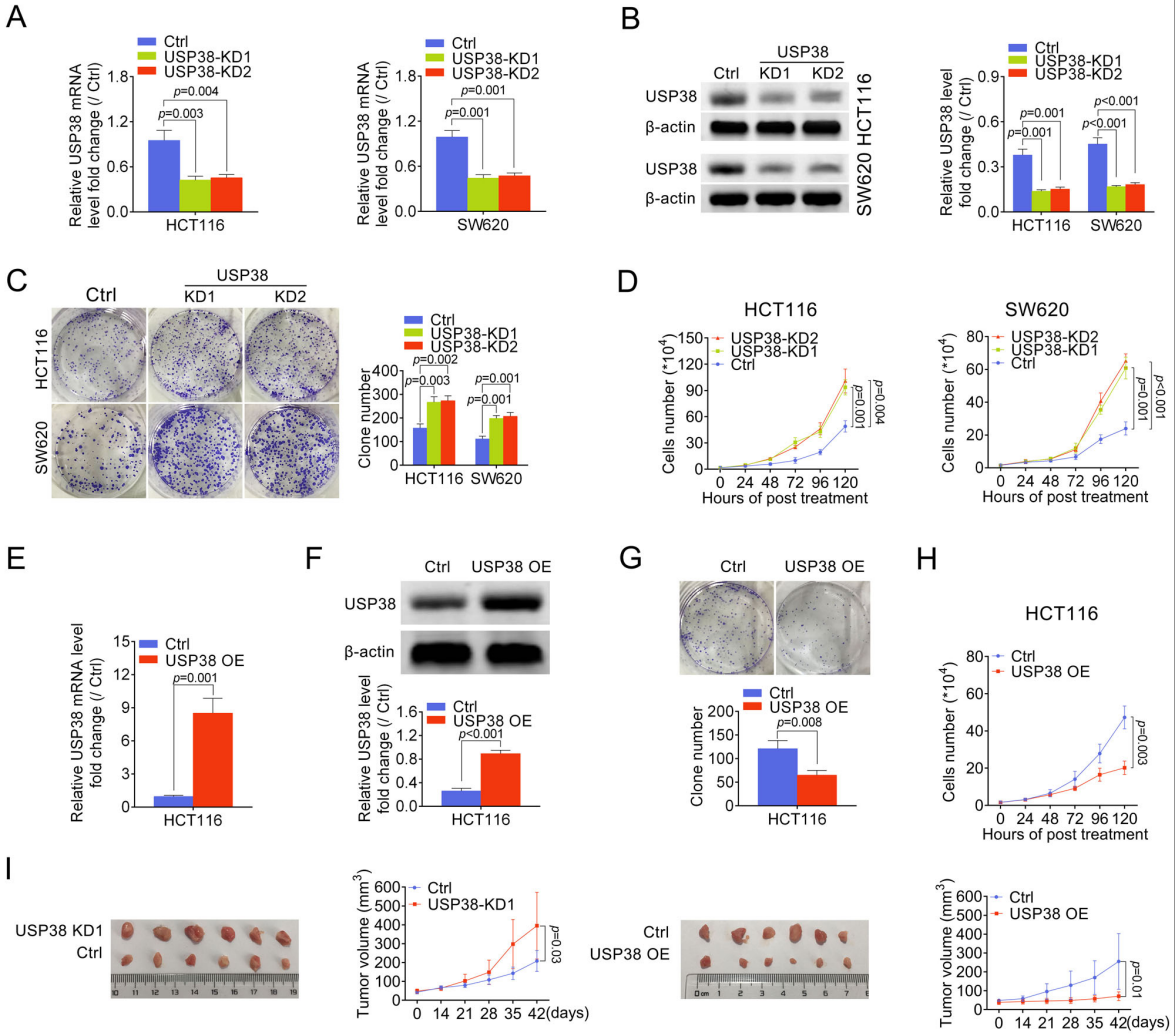
USP38 inhibits colorectal cancer cell growth in vivo and in vitro. **a**, **b** Protein and mRNA levels of USP38 in HCT116 cells or SW620 cells transfected with control shRNA or shRNA targeting USP38. **c** Colonies formed by HCT116 and SW620 cells transfected with control shRNA or shRNA targeting USP38. **d** Number of HCT116 and SW620 cells transfected with control shRNA or shRNA targeting USP38. **e**, **f** Protein and mRNA levels of USP38 in HCT116 cells transfected with control overexpression vector or USP38 overexpression vector. **g** Colonies formed by HCT116 cells transfected with control overexpression vector or USP38 overexpression vector. **h** Number of HCT116 cells transfected with control vector or USP38 overexpression vector. **i** Xenografts generated by HCT116 cells transfected with control shRNA, shRNA targeting USP38, control overexpression vector or USP38 overexpression vector. Lower panels indicated the volume of xenografts generated by HCT116 cells with indicated transfections.

**Fig. 3:**
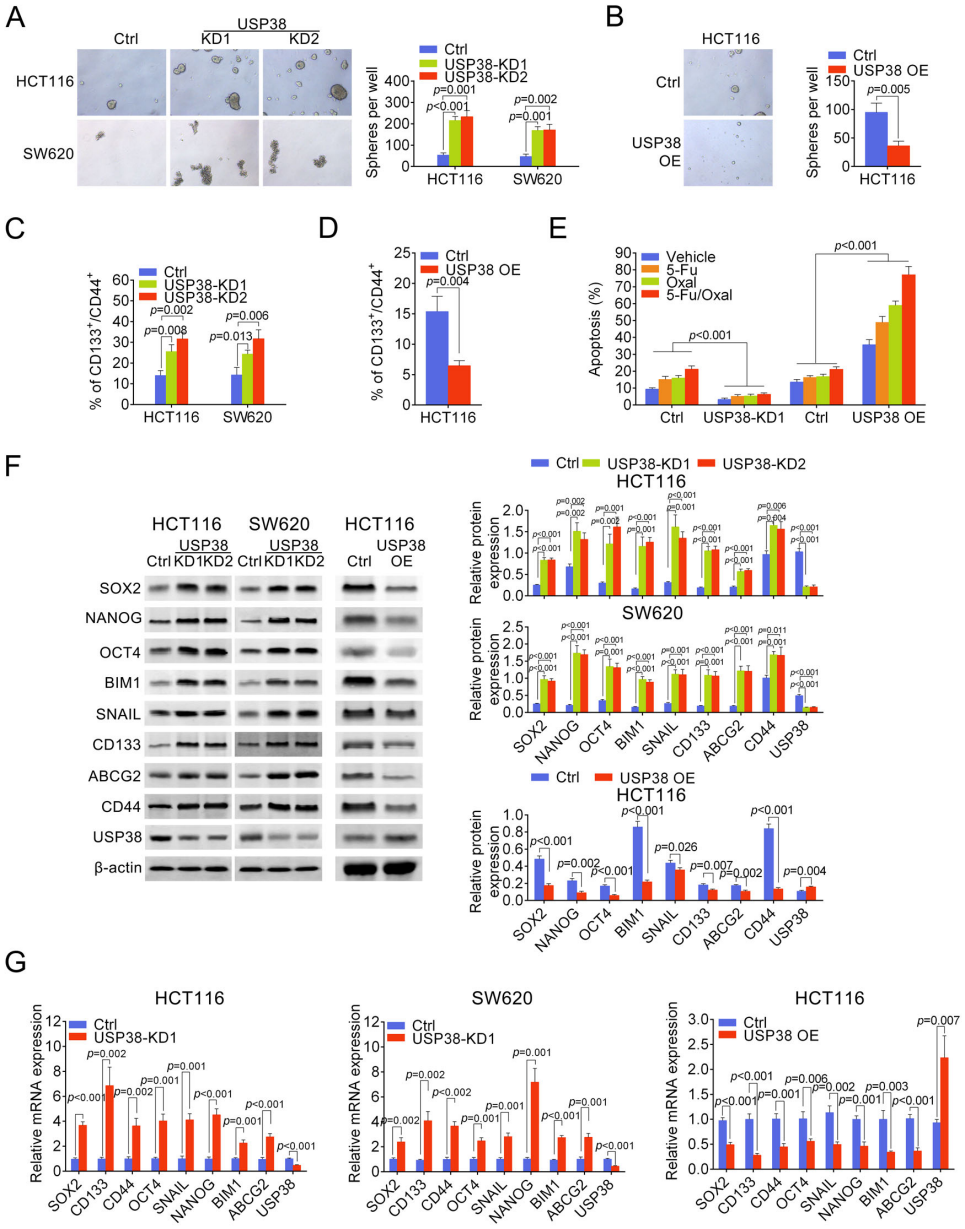
USP38 restrains cancer stem cell population. **a** Typical oncosphere formed by HCT116 or SW620 cells transfected with control shRNA or shRNA targeting USP38. Right panel is quantification of oncospheres. **b** Typical oncosphere formed by HCT116 cells transfected with control overexpression vector or USP38 overexpression vector. Lower panel is quantification of oncospheres. **c** Flow cytometry analysis of CD133 and CD44 double positive cells in HCT116 or SW620 cells transfected with control shRNA or shRNA targeting USP38. **d** Flow cytometry analysis of CD133 and CD44 double positive cells in HCT116 cells transfected with control overexpression vector or USP38 overexpression vector. **e** Flow cytometry analysis of apoptotic HCT116 cells transfected with control shRNA, shRNA targeting USP38, control overexpression vector or USP38 overexpression vector and treated with indicated chemotherapeutics. **f** Protein levels of cancer stem cell-related genes in HCT116 or SW620 cells transfected with control shRNA or shRNA targeting USP38 and HCT116 cells transfected with control overexpression vector or USP38 overexpression vector. Right panels are quantification results. **g** mRNA levels of cancer stem cell related genes in HCT116 or SW620 cells transfected with control shRNA or shRNA targeting USP38 and HCT116 cells transfected with control overexpression vector or USP38 overexpression vector.

To understand how the aberrant USP38 expression affects human colorectal cancer, we next interfered the residual USP38 expression with shRNA (small hairpin RNA) targeting USP38. Both mRNA and protein levels of USP38 were significantly downregulated in HCT116 and SW620 cells with two different shRNA sequences (Fig. 2a, b). We analyzed the colony formation capacity of colorectal cancer cells transfected with control shRNA or shRNA targeting USP38. Our results showed that downregulation of USP38 significantly promoted the colony formation capacity of colorectal cancer cells (Fig. 2c) suggesting that USP38 inhibits the growth of colorectal cancer cells. Moreover, the number of colorectal cancer cells transfected with shRNA targeting USP38 was significantly higher than that of colorectal cancer cells transfected with control shRNA after 120 h of culture (Fig. 3d). Hence, our data indicated that downregulation of USP38 further facilitates the growth of colorectal cancer cells. Next, USP38 was overexpressed in HCT116 cells (Fig. 2e, f). As expected, the number of colonies formed by HCT116 cells and the number of HCT116 cells were significantly decreased when USP38 were overexpressed (Fig. 2g, h). To further validated the tumor suppressor role of USP38 in colorectal cancer cell, we subcutaneously injected HCT116 cells transfected with USP38 shRNA, control shRNA, control vector, and USP38 overexpression vector respectively into nude mice to evaluate the tumorigenesis of colorectal cancer cells with downregulated, normal and upregulated levels of USP38. Importantly, our results showed that downregulation of USP38 significantly facilitated the tumorigenesis of colorectal cancer cells and vice versa (Fig. 2i). Therefore, with both in vitro and in vivo data, we conclude that USP38 inhibits growth of colorectal cancer cells.

### USP38 restrains cancer stem cell population

To dissect the underlying mechanism of how USP38 regulates colorectal cancer cell growth, we analyzed the cancer stem cells properties of colorectal cancer cells with downregulated, normal and upregulated levels of USP38. Oncosphere formation assay showed that downregulation of USP38 significantly facilitated the formation of tumor-spheres while overexpression of USP38 significantly inhibited the formation of tumor-spheres in vitro (Fig. 3a, b). Importantly, flow cytometry analysis of cell surface markers revealed that the number of CD133 and CD44 double positive cells was significantly elevated in colorectal cancer cells transfected with shRNA targeting USP38 and the number of CD133 and CD44 double positive cells was significantly reduced in colorectal cancer cells overexpressing USP38 (Fig. 3c, d). Hence our data suggested that USP38 restrains cancer stem cell population in colorectal cancer. Since cancer stem cell population is critical for chemoresistance, we next examined the responses of colorectal cancer cells with altered USP38 expression to chemotherapeutics. Firstly, we analyzed the number of apoptotic cells in USP38 knock down and USP38 overexpressing HCT116 cells treated with 5-fluorouracil (5-FU), oxaliplatin (Oxal) or 5-FU plus Oxal (5-FU/Oxal). The results showed that 5-FU, Oxal and 5-FU plus Oxal treatments all resulted in increased apoptosis (Fig. 3e). Importantly, downregulation of USP38 significantly reduced the number of apoptotic HCT116 cells treated with chemotherapeutics while upregulation of USP38 significantly consolidated the number of apoptotic HCT116 cells treated with chemotherapeutics (Fig. 3e), suggesting that USP38 sensitize colorectal cancer cells towards chemotherapeutics. Furthermore, we examined the protein levels of cancer stem cell related genes and found that downregulation of USP38 caused significant upregulation of cancer stem cell marker genes SOX2, NANOG, OCT4, BIM1, SNAIL, CD133, ABCG2, and CD44 (Fig. 3f), suggesting that USP38 restrains cancer stem cell population of colorectal cancer cells. Meanwhile, USP38 overexpression resulted in reduction of cancer stem cell marker genes (Fig. 3f) which further indicated that USP38 prohibits stemness of colorectal cancer cells. Additionally, we examined the mRNA levels of these cancer stem cell related genes and found that the transcripts of these genes were significantly decreased in USP38 knockdown cells but elevated in USP38 overexpression cells (Fig. 3g), indicating that USP38 regulates cancer stem cell-related genes at mRNA level.

### USP38 modulates histone acetylation

**Fig. 4:**
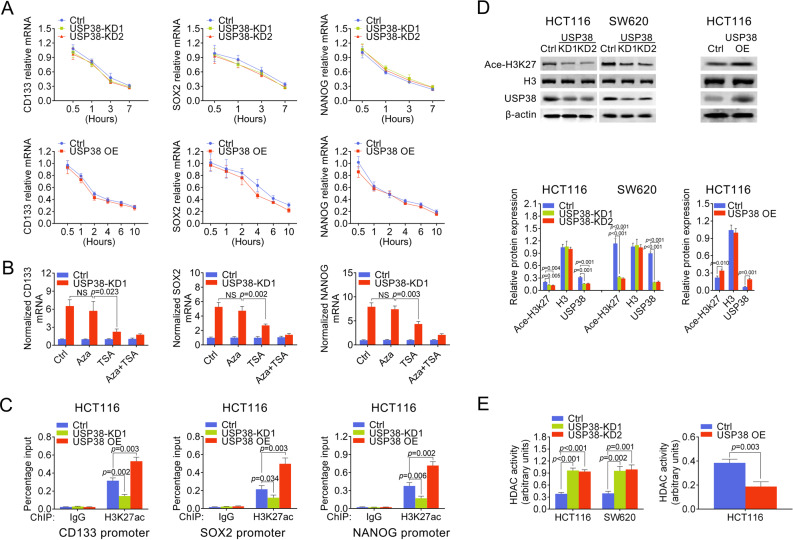
USP38 modulates histone acetylation. **a** mRNA half-life of CD133, SOX2 and NANOG measured in control, USP38 knockdown, and overexpressing HCT116 cells, respectively. **b** mRNA level of CD133, SOX2, and NANOG in control and USP38 knockdown HCT116 cells treated with azacitidine (Aza) or/and Trichostatin A (TSA). **c** Quantitative PCR analysis of H3K27ac chromatin immunoprecipitated CD133, SOX2, and NANOG genes promoter region in control, USP38 knockdown and overexpressing HCT116 cells. **d** Protein levels of acetylated H3K27 and USP38 in control, USP38 knockdown, and overexpressing HCT116 cells, respectively. H3 and β-actin were used as loading control. Lower panels were quantification results. **e** HDAC activities were measured in control, USP38 knockdown and overexpressing HCT116 cells.

Since we found that USP38 suppressed mRNA levels of cancer stem cell related genes, we next analyzed the mRNA half-life of CD133, SOX2, and NANOG to gain further insights into USP38 mediated regulation of gene transcripts. Interestingly, our results showed that the mRNA stability of CD133, SOX2, and NANOG were altered in neither USP38 knockdown cells nor USP38 overexpression cells (Fig. 4a) indicating that USP38 was irresponsible for the mRNA stability of CD133. Hence, we treated wildtype and USP38 knockdown HCT116 cells with azacitidine (Aza) and Trichostatin A (TSA) to evaluate the role USP38 in DNA methylation and histone modification. Surprisingly, our results showed that Aza treatment had no effect on USP38 mediated regulation of genes, while TSA treatment abolished USP38 mediated CD133, SOX2, and NANOG inhibition (Fig. 4b), suggesting that USP38 regulates CD133 via histone acetylation. Hence, we performed chromatin immunoprecipitation (ChIP) against acetylated lysine 27 of histone H3 (H3K27ac) in HCT116 cells with downregulated, normal and upregulated levels of USP38 respectively. Interestingly, we found that downregulation of USP38 resulted in reduced acetylation of H3K27 in the promoter region of these genes and vice versa (Fig. 4c). Moreover, we found that downregulation of USP38 caused significant decrease of overall acetylated H3K27 while USP38 overexpression increased overall levels of acetylated H3K27 (Fig. 4d). Hence, we detected the global histone deacetylase (HDAC) activity in HCT116 cells with downregulated, normal, and upregulated levels of USP38, respectively and found that the global HDAC activities were significantly induced by downregulation of USP38 in HCT116 cells and SW620 cells while the global HDAC activities were inhibited in USP38 overexpressing HCT116 cells (Fig. 4e). Taken together, our data indicated that USP38 modulates histone acetylation via regulating histone deacetylases.

### USP38 upregulates acetylation at H3K27 through deubiquitinating HDAC3

**Fig. 5:**
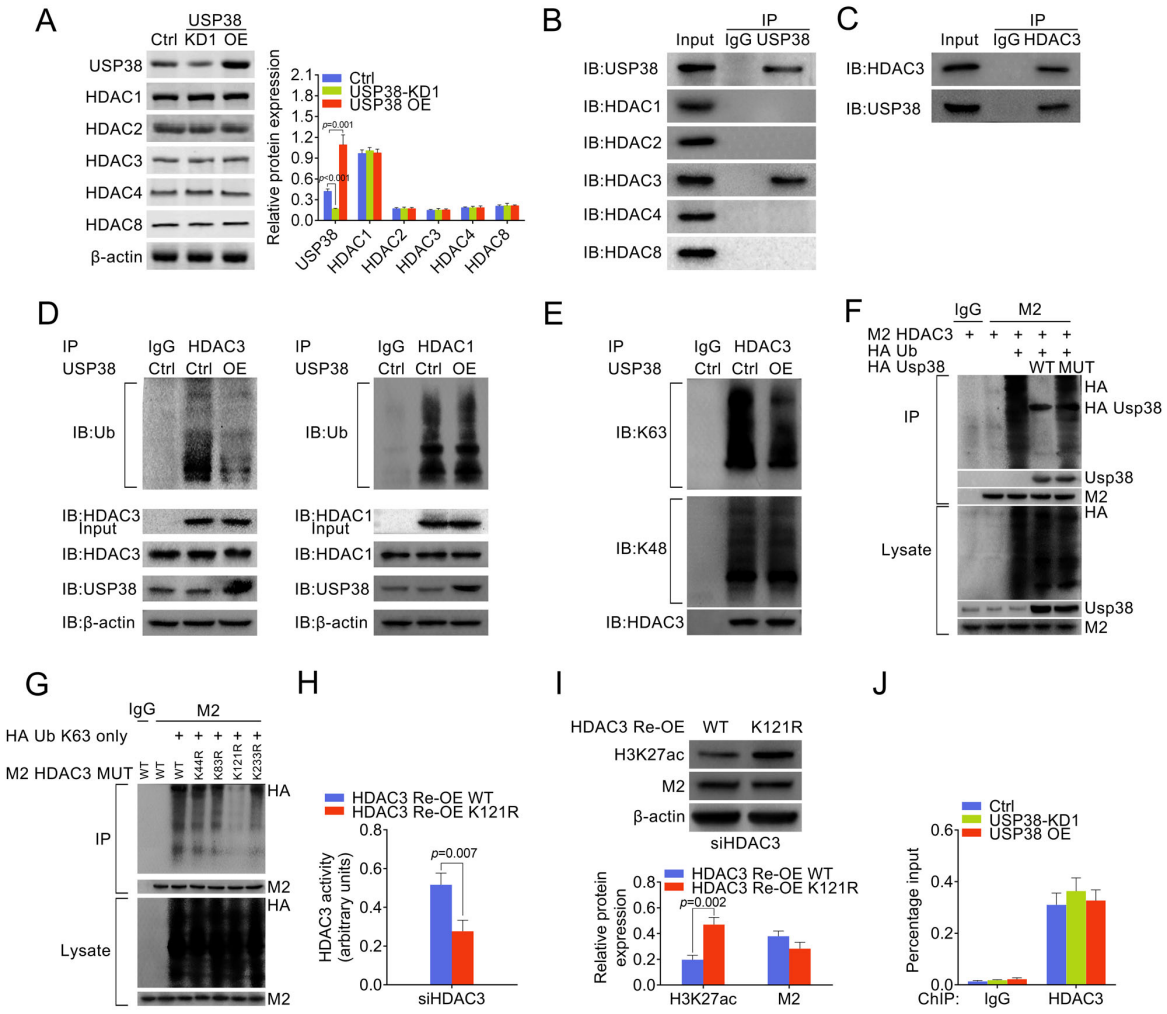
USP38 is a deubiquitinase of HDAC3. **a** Protein levels of HDACs in control, USP38 knockdown and overexpressing HCT116 cells. Right panel is quantification data. **b**, **c** Immunoprecipitation against USP38 and HDAC3 revealed HDAC3 was associated with USP38. **d** Ubiquitination levels of HDAC3 HDAC1 in control or USP38 overexpressing HCT116 cells. β-actin was used as loading control. **e** Ubiquitination levels of K63 chain and K48 chain of HDAC3 in control or USP38 overexpressing HCT116 cells. **f**, **g** Immunoblot analyses of ubiquitinated HDAC3 in HEK293T cells that were transfected with indicated plasmids. Cell lysates were IP with anti-M2 and WB with anti-HA, anti-M2 or anti-USP38. USP38 MUT, a DUB mutant of USP38 (C454S/H857A/D918N). **h** HDAC3 activities were measured in HDAC3 knockdown cells after re-expression of WT HDAC3 or K121R HDAC3 plasmids. **i** Immunoblot analyses of H3K27ac level in HDAC3 knockdown cells after re-expression of WT HDAC3 or K121R HDAC3 plasmids. **j** Anti-HDAC3 antibody was used for ChIP assays in USP38 knockdown and overexpressing cells. QPCR was performed with primers targeting promoter region or CD133.

Next, we analyzed the expression level of HDACs in control, USP38 knockdown, and overexpressing HCT116 cells to determine which HDAC is responsible for USP38 mediated histone modification. However, our results showed that the protein levels of five HDACs (HDAC1, HDAC2, HDAC3, HDAC4, and HDAC8) were not changed in USP38 knockdown and overexpressing cells (Fig. 5a). We then immunoprecipitated (IP) USP38 with HDACs to detect whether any HDAC is associated with USP38. Interestingly, our results showed that HDAC3, but not other HDACs, was specifically associated with USP38 (Fig. 5b, c). Since, USP38 is a potential deubiquitinase, we examined the ubiquitination level of HDAC3 in control and USP38 overexpressing HCT116 cells. Importantly, we found that USP38 overexpression significantly decreased the ubiquitination level of HDAC3 but not HDAC1 and had no effect on the overall protein level of HDAC3 and HDAC1 (Fig. 5d). Moreover, the lysine 63 ubiquitin chain but not the lysine 48 ubiquitin chain of HDAC3 were specifically cleaved by USP38 (Fig. 5e) which explains the unchanged overall HDAC3 protein level. To further prove that USP38 functions as a DUB in removing the K63-linked ubiquitination of HDAC3, we generated a DUB mutant of USP38 (C454S/H857A/D918N), which lacks the DUB activity^16^. We found that the ubiquitination of HDAC3 was erased by WT USP38 but not the enzyme activity mutant of USP38 (Fig. 5f). In order to identify the K63 ubiquitination site on HDAC3, we generated four K to R mutants of HDAC3 (K44R, K83R, K121R, and K233R). WT HDAC3 and four KR mutant HDAC3 were transfected with HA-Ub K63 only (all lysine sites on Ubiquitin were mutated to arginine, except for lysine at 63, the HA-Ub K63 only plasmid could only generate Lys-63 ubiquitination chain) plasmids. We found that K121R mutant of HDAC3 was unable to form K63 ubiquitination chain, suggesting that lysine 121 may be the K63 ubiquitination site on HDAC3 (Fig. 5g). Then, we detected HDAC3 activity in HDAC3 depleted cells after reconstructed HDAC3 expression with WT or K121R mutant HDAC3. Our results showed that HDAC3 K121R mutant cells exhibited lower HDAC3 activity compared with WT HDAC3 (Fig. 5h). K121R mutant also showed higher level of H3K27ac compared with WT HDAC3 (Fig. 5i). These results indicated that K63 ubiquitination of HDAC3 was required for the deacetylation activity of HDAC3. Next, we performed a ChIP assay to illustrate the binding infinity of HDAC3 and the promoter of CD133. As shown in Fig. 5j, the binding of HDAC3 to the promoter of CD133 was affected by neither USP38 knockdown nor USP38 overexpression. Taken together, K121 of HDAC3 is required for its K63-linked ubiquitination of HDAC3 and the deacetylation activity of HDAC3. Hence, our data indicated that USP38 functions as a deubiquitinase of HDAC3 to modulate the acetylation of histones and regulate gene expression.

**Fig. 6:**
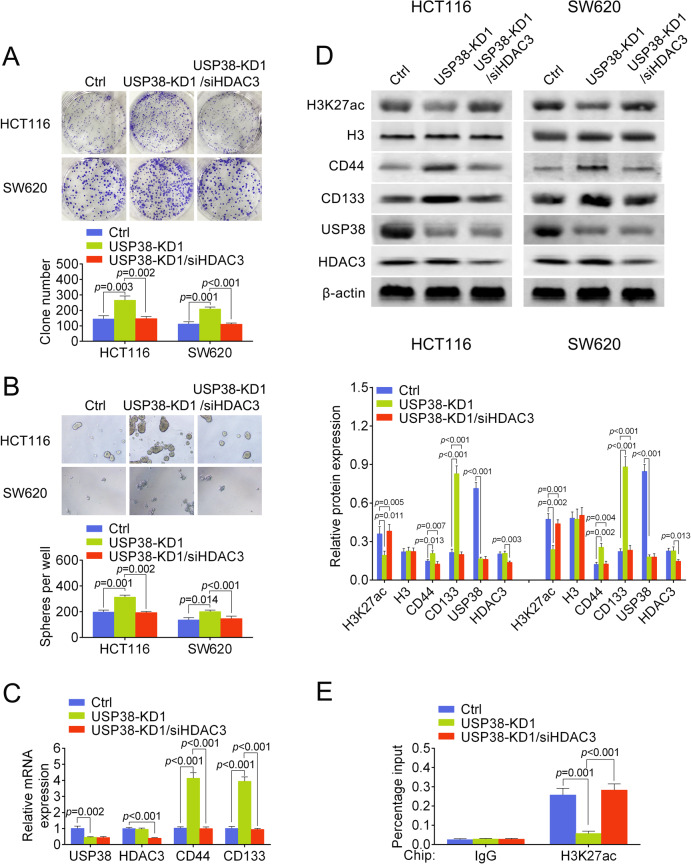
HDAC3 is functionally involved in USP38 mediated gene regulation. **a** Colonies formed by HCT116 and SW620 cells transfected with control shRNA, shRNA targeting USP38 and shRNA targeting USP38 plus siRNA targeting HDAC3. Lower panel is quantification results. **b** Oncospheres formed by HCT116 and SW620 cells transfected with control shRNA, shRNA targeting USP38 and shRNA targeting USP38 plus siRNA targeting HDAC3. Lower panel is quantification results. **c** mRNA levels of USP38, HDAC3, CD44, and CD133 in HCT116 cells transfected with control shRNA, shRNA targeting USP38 and shRNA targeting USP38 plus siRNA targeting HDAC3. **d** Protein levels of H3K27ac, USP38, HDAC3, CD44, and CD133 in HCT116 cells transfected with control shRNA, shRNA targeting USP38 and shRNA targeting USP38 plus siRNA targeting HDAC3. H3 and β-actin were used as loading control. Lower panels were quantification results. **e** Quantitative PCR analysis of H3K27ac chromatin immunoprecipitated CD133 promoter region in control, USP38 knockdown and USP38 and HDAC3 double knockdown HCT116 cells.

To validate that HDAC3 is functionally involved in USP38 mediated tumor suppression, HDAC3 was further interfered in USP38 knockdown cells. Our results showed that downregulation of HDAC3 attenuated the increased colony formation and tumor-sphere formation capability induced by downregulation of USP38 (Fig. 6a, b). Moreover, induction of CD44 and CD133 mRNA by USP38 knockdown were also attenuated by further knockdown of HDAC3 (Fig. 6c). In both HCT116 and SW620 cells, simultaneous knockdown of USP38 and HDAC3 attenuated the decreased H3K27ac level and increased CD44 and CD133 (Fig. 6d). Importantly, the decreased acetylation level of H3K27 in the promoter region of CD133 in USP38 knockdown cells was rescued by additional knockdown of HDAC3 (Fig. 6e). Taken together, our data demonstrated that HDAC3 is functional responsible for USP38 mediated histone modifications, which further controls the expression of cancer stem cell-related genes.

### Clinical significance of USP38 in colorectal cancer patients

**Fig. 7:**
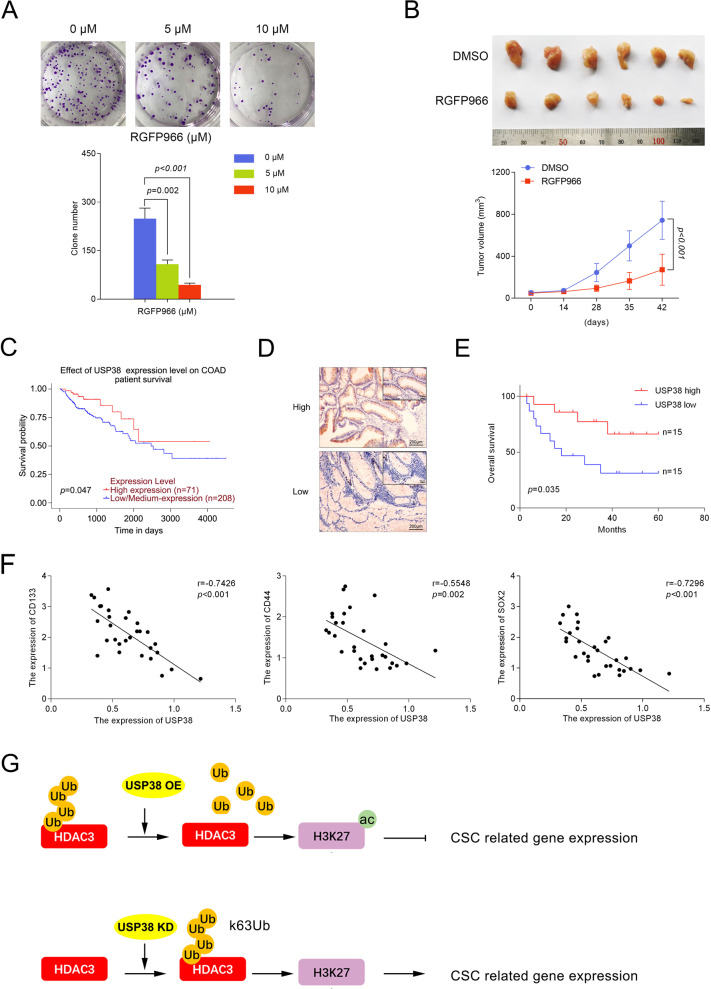
Clinical significane of USP38 in colorectal cancer patient. **a** HCT116 cells were treated with indicated concentration of RGFP966 for 10 days. Clonogenic survival assay were performed. **b** HCT116 cells were implanted subcutaneously into the flank regions of nude mice. When the tumors reached 100 mm^3^, RGFP966 treatment was initiated. Each group consisted of six animals. Tumor volumes were measured every week. Mice were euthanized after 42 days, and the growth curve were plotted. **c** Survival probability of patients with high USP38 expression (red line) or low USP38 expression (blue line). COAD stands for colorectal adenocarcinoma. **d** Characterization of high USP38 expression and low USP38 expression based on IHC results. **e** Overall survival of colorectal cancer patients with high USP38 expression (red line) or low USP38 expression (blue line). **f** Expression correlation between USP38 and CD133, CD44, and SOX2. **g** Cartoon illustration of USP38 mediated histone modification and downstream gene regulation.

Since HDAC3 plays a critical role in regulation of the expression of cancer stem cell related genes, we used the selective inhibitor of HDAC3 RGFP966 to investigate the function of USP38-HDAC3 in colorectal cancer cell growth. We found that RGFP966 treatment significantly decreased the number of colonies in HCT116 cells (Fig. 7a). To investigate the effect of RGFP966 on colorectal cancer cell growth in vivo, we treated mice bearing subcutaneous tumors generated from HCT116 cell with DMSO or RGFP966. HDAC3 inhibition by RGFP966 had a significan slower rate of growth of HCT116 derived tumors as compared with DMSO (Fig. 7b).

Since our data demonstrated a tumor suppressor role of USP38 in colorectal cancer patients, we then analyzed clinical samples to validate the clinical significance of USP38 in colorectal cancer patients. First of all, we analyzed the survival probability with online database (http://ualcan.path.uab.edu/cgi-bin/TCGA-survival1.pl?genenam=USP38&ctype=COAD) and found that high USP38 expression level indicated better survival probability of colorectal cancer patients (Fig. 7c). We collected 30 patient samples and divided the patients into two groups based on the expression level of USP38 (Fig. 7d). Based on the survival information, we plotted the overall survival rates of enrolled patients (Fig. 7e). More importantly, we found that the expression of USP38 was negatively correlated with the expression of CD133, CD44, and SOX2 (Fig. 7f) suggesting that USP38 negatively regulates cancer stem cells in colorectal cancer patients.

## Discussion

Taken together, our data demonstrated that USP38 is a specific deubiquitinase of K63 ubiquitin chain of HDAC3. Downregulation of USP38 led to ubiquitination of HDAC3, which resulted in decreased acetylation levels of H3K27 in the promoter regions of cancer stem cell related genes. Finally, oncogenes are transcribed and promotes tumorigenesis of colorectal cancer (Fig. 7e).

Deubiquitinases have been actively involved in tumorigenesis via regulating cancer stem cells^20^. Our work revealed that USP38 is a novel player in regulating stemness of colorectal cancer. Our results showed that stemness markers like SOX2, NANOG, OCT4, and BIM1 were significantly induced by inhibition of USP38 at both mRNA and protein levels. Importantly, we demonstrated that the cellular level of USP38 regulates the histone modification status of these cancer stem cell-regulated genes. This promising role of HDAC3 in regulating cancer stem cell related genes provided novel information on chemoresistance of human colorectal cancer. Previouse reported showed that HDAC3 plays crucial roles in cancer stem cells via genome wide epigenetic modifications^21,22^. Moreover, HDAC3 inhibition facilitated non-small cell lung cancer in overcoming osimertinib resistance^23^. Taken together, these data have demonstrated a potential role of HDAC3 inhibitors for chemoresistance overcoming. It has been proposed that Argonaut family protein PIWIL2 interacts with and stabilize HDAC3 from ubiquitin-mediated degradation by competitive association with E3 ubiquitin ligase Siah2^14^. Since PIWI proteins were also critical regulators in stem cells and cancer cells^24,25^, it is of importance to evalue the interaction between these RNA binding proteins and histone deacetylases to identify sequence specific mechanisms of histone modifications.

Meanwhile, reports have shown that deubiquitinases are also critical regulators of histone modifications^26,27^. Here, our results showed that USP38 is a key regulator of HDAC3 in a posttranslational manner and ubiquitination levels of HDAC3 determines the acetylation of histones of specific genes. Interestingly, deubiquitinating enzyme can be regulated by histone deacetylase inhibitors^27^. It has been shown that USP38 is required for the deubiquitination of LSD1, the key factor of several histone deacetylase complexes^28^. In addition, LSD1 and HDAC3 cooperated with each other to modulate signal transducer and activator of transcription 5 (STAT5) dependent transcriptional regulation^29^. These data suggested the involvement of LSD1 in USP38 mediated HDAC3 ubiquitination and regulations of downstream target genes. Previous report showed that USP38 specifically cleaves K33 ubiquitin chains from TBK1. The deubiquitination process further allows subsequent K48 ubiquitination mediated by deltex E3 ubiquitin ligase 4 (DTX4) and TRAF interacting protein (TRIP)^15^. It is of importance to analyze the exact lysine on which HDAC3 was ubiquitinated and whether the deubiquitination mediated by USP38 is followed by other types of modification which facilitates HDAC3 mediated deacetylation. Moreover, it has been shown that USP38 directly associated with JunB and deubiquitinated K48 poly-ubiquitination of JunB in Th2-mediated allergic asthma^16^. Whether USP38 plays roles in tumor immunity requires further explorations. Moreover, studies have demonstrated critical roles of deubiquitinases in protein homeostasis^30,31^. Our data showed that HDAC3 protein level was merely affected by either knockdown or overexpression of USP38. Therefore, it is of potential to further dissect the ubiquitination status of HDAC3 to further analyze the function of HDAC3.

Our work revealed a novel tumor suppressor function of USP38 in human colorectal cancer via directly regulating ubiquitination status of HDAC3. These results enrich the epigenetic nexus, which facilitate the carcinogenesis and chemoresistance mediated by cancer stem cells. Hence, targeting USP38 as well as HDAC3 are promising strategy in overcoming chemoresistance of colorectal cancer. Moreover, we have provided information on the expression level of USP38 in different stages of colorectal cancer and showed that high level of USP38 indicated a better overall survival suggesting that USP38 may have the potential of serving as a diagnostic marker for colorectal cancer staging.

## Data Availability

All data generated or analyzed during this study are included in this published article.

## References

[1] Vymetalkova V, Vodicka P, Vodenkova S, Alonso S, Schneider-Stock R (2019). DNA methylation and chromatin modifiers in colorectal cancer. Mol. Asp. Med..

[2] Bian S (2018). Single-cell multiomics sequencing and analyses of human colorectal cancer. Science.

[3] Sun JY (2017). Marine-derived chromopeptide A, a novel class I HDAC inhibitor, suppresses human prostate cancer cell proliferation and migration. Acta Pharmacol. Sin..

[4] Wang Q (2019). Elevating H3K27me3 level sensitizes colorectal cancer to oxaliplatin. J. Mol. Cell Biol..

[5] Ye C (2018). Inhibition of histone deacetylase 7 reverses concentrative nucleoside transporter 2 repression in colorectal cancer by up-regulating histone acetylation state. Br. J. Pharmacol..

[6] Qin, J., Wen, B., Liang, Y., Yu, W. & Li, H. Histone modifications and their role in colorectal cancer (Review). *Pathol. Oncol. Res.* 1–11 (2019). 10.1007/s12253-019-00663-8 PMC7471167 31055775

[7] Haigney A, Ricketts MD, Marmorstein R (2015). Dissecting the molecular roles of histone chaperones in histone acetylation by type B histone acetyltransferases (HAT-B). J. Biol. Chem..

[8] Yang WM, Yao YL, Sun JM, Davie JR, Seto E (1997). Isolation and characterization of cDNAs corresponding to an additional member of the human histone deacetylase gene family. J. Biol. Chem..

[9] Wilson AJ (2006). Histone deacetylase 3 (HDAC3) and other class I HDACs regulate colon cell maturation and p21 expression and are deregulated in human colon cancer. J. Biol. Chem..

[10] Weichert W (2008). Class I histone deacetylase expression has independent prognostic impact in human colorectal cancer: specific role of class I histone deacetylases in vitro and in vivo. Clin. Cancer Res..

[11] Wang X (2016). CBX4 suppresses metastasis via recruitment of HDAC3 to the Runx2 promoter in colorectal carcinoma. Cancer Res..

[12] Moseley VR, Morris J, Knackstedt RW, Wargovich MJ (2013). Green tea polyphenol epigallocatechin 3-gallate, contributes to the degradation of DNMT3A and HDAC3 in HCT 116 human colon cancer cells. Anticancer Res..

[13] Zhao HL, Ueki N, Hayman MJ (2010). The Ski protein negatively regulates Siah2-mediated HDAC3 degradation. Biochem. Biophys. Res. Commun..

[14] Zhang Y (2018). PIWIL2 suppresses Siah2-mediated degradation of HDAC3 and facilitates CK2alpha-mediated HDAC3 phosphorylation. Cell Death Dis..

[15] Lin M (2016). USP38 inhibits type I interferon signaling by editing TBK1 ubiquitination through NLRP4 signalosome. Mol. Cell.

[16] Chen S (2018). USP38 critically promotes asthmatic pathogenesis by stabilizing JunB protein. J. Exp. Med..

[17] Liu W, Zhang Q, Fang Y, Wang Y (2018). The deubiquitinase USP38 affects cellular functions through interacting with LSD1. Biol. Res..

[18] Chen X (2016). Bcl-3 regulates TGFbeta signaling by stabilizing Smad3 during breast cancer pulmonary metastasis. Cell Death Dis..

[19] Liang Y (2015). Epigenetic activation of TWIST1 by MTDH promotes cancer stem-like cell traits in breast cancer. Cancer Res..

[20] Lei H, Shan H, Wu Y (2017). Targeting deubiquitinating enzymes in cancer stem cells. Cancer Cell Int..

[21] Peng Z, Zhou W, Zhang C, Liu H, Zhang Y (2018). Curcumol controls choriocarcinoma stem-like cells self-renewal via repression of DNA methyltransferase (DNMT)- and histone deacetylase (HDAC)-mediated epigenetic regulation. Med. Sci. Monit..

[22] Hsieh HY (2017). Targeting breast cancer stem cells by novel HDAC3-selective inhibitors. Eur. J. Med. Chem..

[23] Tanimoto A (2017). Histone deacetylase 3 inhibition overcomes BIM deletion polymorphism-mediated osimertinib resistance in EGFR-mutant lung cancer. Clin. Cancer Res..

[24] Wang C (2019). Heat shock protein DNAJA1 stabilizes PIWI proteins to support regeneration and homeostasis of planarian Schmidtea mediterranea. J. Biol. Chem..

[25] Chen H, Xu Z, Liu D (2019). Small non-coding RNA and colorectal cancer. J. Cell. Mol. Med..

[26] He M (2019). Intrinsic apoptosis shapes the tumor spectrum linked to inactivation of the deubiquitinase BAP1. Science.

[27] Wu X (2019). MGMT-activated DUB3 stabilizes MCL1 and drives chemoresistance in ovarian cancer. Proc. Natl Acad. Sci. USA.

[28] Yang Y (2018). LSD1 coordinates with the SIN3A/HDAC complex and maintains sensitivity to chemotherapy in breast cancer. J. Mol. Cell Biol..

[29] Nanou A (2017). The dual role of LSD1 and HDAC3 in STAT5-dependent transcription is determined by protein interactions, binding affinities, motifs and genomic positions. Nucleic Acids Res..

[30] Harris IS (2019). Deubiquitinases maintain protein homeostasis and survival of cancer cells upon glutathione depletion. Cell Metab..

[31] Chen X (2019). USP17 suppresses tumorigenesis and tumor growth through deubiquitinating AEP. Int. J. Biol. Sci..

